# Involvement of Peripheral Serotonin in Blood Cells in Healthy Cyclical Mares of Different Ages

**DOI:** 10.3390/vetsci12060548

**Published:** 2025-06-04

**Authors:** Katiuska Satué, Deborah La Fauci, Pietro Medica, María Gemma Velasco-Martínez, Giuliana Barbiera, Esterina Fazio

**Affiliations:** 1 Department of Animal Medicine and Surgery, Faculty of Veterinary Medicine, CEU-Cardenal Herrera University, Tirant lo Blanc, 7, Alfara del Patriarca, 46115 Valencia, Spain; ksatue@uchceu.es; 2 Department of Veterinary Sciences, Veterinary Physiology Unit, Polo Universitario Annunziata, Via Palatucci 13, 98168 Messina, Italy; pmedica@unime.it (P.M.); mariagemma.velasco@alumnos.uchceu.es (M.G.V.-M.); fazio@unime.it (E.F.); 3 Pharmaceutical and Chemical Technician, 98168 Messina, Italy; giulianabarbiera@gmail.com

**Keywords:** age, 5-HT, immune cells, cyclic mares

## Abstract

The estrous cycle involves hormonal fluctuations that influence serotonin (5-HT) concentrations and immune cell activity. During the follicular phase, estrogen increases 5-HT synthesis, enhancing cellular responses to inflammation. In contrast, in the luteal phase, progesterone suppresses immune function, reducing 5-HT availability. Age-related changes in hormone concentrations can affect these processes, potentially altering cellular responses and 5-HT dynamics in the oldest females. This study hypothesizes the relationship between 5-HT and blood cells in healthy cyclic mares, considering age as an influencing factor. Understanding these interactions offers insights into reproductive health and immune regulation in equine species. The aim was to describe the interaction between 5-HT and blood cells in twenty-five healthy cyclic Spanish Purebred mares, evaluating the effect of aging. For this purpose, the mares were classified into two age groups as follows: from 4 to 9 years (*n* = 12 younger mares) and from 10 to 15 years (*n* = 13 older mares). Younger and older mares showed an opposite 5-HT trend at +5 and +16 day of cycle (d), with greater concentrations in younger mares (*p* < 0.05) and lower values in older mares (*p* < 0.05) than at −5 and 0 d. Older mares showed a lower 5-HT concentration at +5 and +16 d (*p* < 0.05) than younger mares. Mares of both ages showed a superimposed WBC trend, with the greatest number both at −5 and 0 d (*p* < 0.05). Older mares showed a lower WBC number at −5 d (*p* < 0.05), and lower NFS and LYMPH numbers both at +5 and +16 d (*p* < 0.05) than younger mares; the latter showed the lowest NFS number at +16 d, and LYMPH number at +5 and +16 d. Mares of both ages showed a similar trend for the N/L ratio. Older mares showed a lower MON number at +5 d (*p* < 0.05), greater PLTs at +5 and +16 d (*p* < 0.05) than younger mares, and a greater PLT number at −5 and 0 d than at +5 and +16 d (*p* < 0.05). The activity of 5-HT is regulated by its rate of synthesis, release, and metabolism according to age. Aging reduced the 5-HT concentrations and the number of WBCs, NFSs, LYMPHs, and MONs, inducing correlations among these and 5-HT in healthy young and old cyclic mares.

## Simple Summary

Systemic 5-hydroxytryptamine or serotonin (5-HT) affects immune regulation also during the estrous cycle. The aim was to assess the bidirectional interaction between 5-HT and immune cell reactions in twenty-five healthy cyclic Spanish Purebred mares, evaluating the aging effect. Older mares showed lower 5-HT concentrations at +5 and +16 days (d) (*p* < 0.05) than younger mares. Mares of both ages showed a superimposed white blood cell (WBC) trend, with the greatest number at −5 and 0 d (*p* < 0.05). Older mares showed a lower WBC number at −5 d (*p* < 0.05), and a lower number of neutrophils (NFSs) and lymphocytes (LYMPHs) at both +5 and +16 d (*p* < 0.05) than younger mares. Mares of both ages showed a similar trend for the N/L ratio. Older mares exhibited a lower number of monocytes (MONs) at +5 d (*p* < 0.05), and greater platelets (PLTs) at +5 and +16 d (*p* < 0.05) than younger mares. The activity of 5-HT is regulated by its rate of synthesis, release, and metabolism according to age. Aging reduced the 5-HT concentrations and the number of WBCs, NFSs, LYMPHs, and MONs, inducing correlations among these parameters and 5-HT in cyclic mares of different ages.

## 1. Introduction

Serotonin or 5-hydroxytryptamine (5-HT) is a neurotransmitter that is well known for its role in the central nervous system, but it also plays key roles in the regulation of hemostasis and immune responses through its action on blood cells. In females, 5-HT concentrations and its activity in platelets (PLTs), neutrophils (NFSs), monocytes (MONs), and lymphocytes (LYMPHs) fluctuate throughout the estrous cycle, influenced by hormonal variations, particularly estrogen and progesterone concentrations [1,2,3].

Notably, 5-HT plays a pivotal role in PLT function, particularly in aggregation and hemostasis. PLTs store large amounts of 5-HT in dense granules, which they release upon activation to enhance vasoconstriction and PLT recruitment. The responsiveness of PLTs to 5-HT is modulated by hormonal fluctuations during the estrous cycle. Indeed, high estrogen concentrations during the follicular phase have been shown to increase the expression of 5-HT receptors—particularly 5-HT_2_A—on PLTs, potentially enhancing their sensitivity to serotonergic stimuli and promoting aggregation and clot formation [4]. In contrast, the luteal phase, characterized by elevated progesterone (P4) and reduced estrogen concentrations, may suppress serotonergic signaling, reducing PLT reactivity [5]. These cyclical variations suggest that the 5-HT activity of PLTs is not constant but is closely linked to the hormonal environment, which may have implications for healthy coagulation and vascularization across different phases of the estrous cycle [6].

The function of neutrophils (NFSs), which are key cells in the innate immune response, is influenced by 5-HT. These leukocytes express serotonergic receptors such as 5-HT_1_A, 5-HT_2_A, and 5-HT_3_, as well as the 5-HT transporter (SERT), enabling them to respond to serotonergic signals. Furthermore, 5-HT is involved in the recruitment of NFSs to sites of inflammation, acting as a PLT-derived chemokine [7,8].

MONs express various subtypes of 5-HT receptors, including 5-HT_1_A, 5-HT_2_A, 5-HT_3_, 5-HT_4_, and 5-HT_7_, as well as the 5-HT transporter (SERT) and enzymes responsible for its synthesis and degradation. Furthermore, 5-HT modulates the function of these phagocytes by influencing cytokine secretion. These interactions suggest that 5-HT plays a role in regulating both the innate and adaptive immune response [8,9].

T and B LYMPHs, as well as natural killer (NK) cells, express several subtypes of 5-HT receptors, including 5-HT_1_A, 5-HT_2_A, 5-HT_3_, 5-HT_7_, and 5-HT_4_, indicating their ability to respond to serotonergic signals. Additionally, 5-HT can influence LYMPH activation and cytokine production, affecting both innate and adaptive immunity. For example, 5-HT may induce T cell activation and cytokine release in response to specific stimuli [8].

Hormonal fluctuations modulate the expression of serotonergic receptors and transporters in blood cells, affecting their function and contributing to variability in immune responses and coagulation during the different phases of the estrous cycle [1,2,3]. During the estrous cycle, hormonal fluctuations may alter the sensitivity of MONs to 5-HT, modulating their response to inflammatory stimuli [8,9]. In addition, estrogen enhances LYMPH presence and function, whereas P4 tends to suppress proinflammatory immune responses [8,10].

With advancing age, alterations in serotonergic signaling have been observed. Ferlazzo et al. [11] investigated the influence of age on plasma 5-HT concentrations in Thoroughbred horses. The study found that both plasma 5-HT and tryptophan concentrations were significantly greater in foals compared to adult horses, suggesting a different rate of 5-HT synthesis that is correlated with age. Recently, it was shown that age modulates, in a significant manner, the physiological changes in hematological variables of healthy Spanish Purebred horses, establishing reference values, with a decline in the number of white blood cells (WBCs), and platelets (PLTs) in mares according to their age, indicating a decrease in the narrow bone response [12].

To the authors’ knowledge, studies on changes in immune cells during the cycle have been reported in women [13,14,15,16] and in bitches [14,15,16,17,18], providing controversial results, although they are not related to 5-HT concentrations.

In light of our previous studies and the pertinent literature data, we provide evidence for the consistent role of serotoninergic activity in the follicular fluid (FF) of mares, and the essential role of 5-HT in steroidogenesis during dominant follicle growth; we place a special emphasis on its existence in the FF of the dominant follicle, and an interaction with steroids was also observed in equine species [19]. Indeed, 5-HT plays a crucial role in ovulation by influencing immune cell functions for cells such as NFSs, LYMPHs, and MONs that express 5-HT receptors and participate in the inflammatory processes necessary for oocyte release [20]. Inflammation facilitates the maturation of follicles and the release of oocytes through a series of coordinated immune responses [21].

Based on this evidence, our aim for conducting this study was to evaluate the possible relationship between 5-HT and blood cells, with special attention paid to the number of WBCs, NSFs, LYMPHs, NBMs, and PLTs, and to the neutrophil/lymphocyte ratio (N/L) at different times during the estrous cycle in healthy mares, assessing the effect of different ages. Since ovulation is characterized by an inflammatory state and age modifies the 5-HT profile, the objective of this study was to evaluate the possible interactions and variations in blood cells in healthy younger and older mares of 4–9 and 10–15 years, respectively, during the periovulatory period. These possible variations can underscore the importance of considering the timing of the cycle and age in clinical evaluations related to the physiological effects of 5-HT.

## 2. Materials and Methods

### 2.1. Animals

All methods and procedures used in this study are in line with the Spanish law (RD 37/2014) guidelines that regulates the protection of animals used for scientific purposes. The Animal Ethics Committee for the Care and Use of Animals of the CEU-Cardenal Herrera University (Spain) concluded that the experimental proposed study did not need an ethical approval, since this experiment was part of the clinical evaluation of the animals at this stage of their cycle (CEEA 22/01). Twenty-five healthy cyclic mares were included in the present study, which were distinguished according to age into two groups as follows: from 4 to 9 years (*n* = 12 younger mares) and from 10 to 15 years (*n* = 13 older mares).

The mares’ inclusion criteria were the following: (i) the presence of physiological cyclicity during the previous breeding seasons; (ii) the absence of reproductive pathologies or evidence of disease; and (iii) the absence of treatment with antibiotics or anti-inflammatory drugs within a month of the start of the study. The mean inter-estrus interval was paired to 21.8 ± 1.5 days, and the estrous cycle length was 22.2 ± 0.13 days. Management practices and feeding were the same for all mares studied. They were fed twice daily, with 2–3 kg of concentrate, 2–3 kg of alfa-alfa hay, and wheat straw, and water was provided ad libitum. Mare’s estrus was detected in the presence of the stallion and by transrectal ultrasonography of the uterus and ovaries. Mares in heat were receptive to the stallion, had uterine edema, and had a follicle 35 mm in diameter or greater in the ovary. To monitor enlargement of the preovulatory follicle, and the presence of uterine edema, and to predict ovulation time, transrectal ultrasonography using a 5 MHz probe (Sonosite 180 Plus) was performed.

### 2.2. Blood Samples

Blood samples were collected under ultrasound guidance according to the following protocol: 5 days before ovulation, on day 0 when the preovulatory follicle reached its maximum diameter and ovulation occurred, and on days +5 and +16 post-ovulation. Samples were collected by jugular venipuncture using 20 mL disposable syringes with a Luer cone (Becton Dickinson Discardit^®^ II; BD San Agustin de Guadalix, Madrid, Spain) and 18–20 G needles (Sterican^®^, Braun Melsungen AG, Melsungen, Germany). After collection, samples were transferred to EDTA tubes (Tapval^®^; Acralab SLU, Alicante, Spain) and glass tubes containing coagulation activators (Tapval^®^; Acralab SLU Alicante, Spain). Samples stored in EDTA were analyzed immediately after collection, and the samples stored with coagulation accelerators were centrifuged at 3000× *g* for 10 min (P Selecta^®^ centrifuge; LabNet Biotécnica, Madrid, Spain), storing the serum at −20 °C until further analysis.

### 2.3. Analytical Procedures

An enzyme-linked immunosorbent assay (ELISA) (DLD Diagnostics GmbH) was used to measure 5-HT (ng/mL). The monoclonal antibody used to determine this parameter is cross-reactive with serotonin (100%), tryptamine (1.3%), 5-methoxytryptamine (0.18%), and melatonin (<0.013%). This technique has been previously validated and used in horses [22].

White blood cell (WBC), lymphocyte (LYMPH), neutrophil (NFS), monocyte (MON), and platelet (PLT) counts (G/L) were performed using ADVIA 2020i, Erlangen, Germany. The neutrophil/lymphocyte ratio (N/L ratio) was also calculated. Cell morphological analysis was performed on blood films using the Diff-Quick rapid panoptic method (Gailand Chemical Co., Flintshire, UK).

### 2.4. Statistical Analysis

Descriptive statistics (mean, median, and standard deviation, as well as minimum and maximum values) were calculated for each variable. Age comparison using the Student’s *t*-test was analyzed. The Mann–Whitney comparative test was used for data without a normal distribution. To analyze the effect of age, a one-way analysis of variance was used, before logarithmic data transformation, to meet the conditions of normality and homoscedasticity using the Kolmogorov–Smirnov test. In those cases in which this analysis showed statistically significant differences, a Tukey HSD test was then applied. For variables not normally distributed, a Kruskal–Wallis comparative test was used. When the differences were statistically significant, comparisons between groups were made using the Mann–Whitney test with Bonferroni adjustment. Using multivariate regression, the combined effect of age was analyzed. The differences were considered statistically significant when *p* < 0.05.

## 3. Results

**Figure 1:**
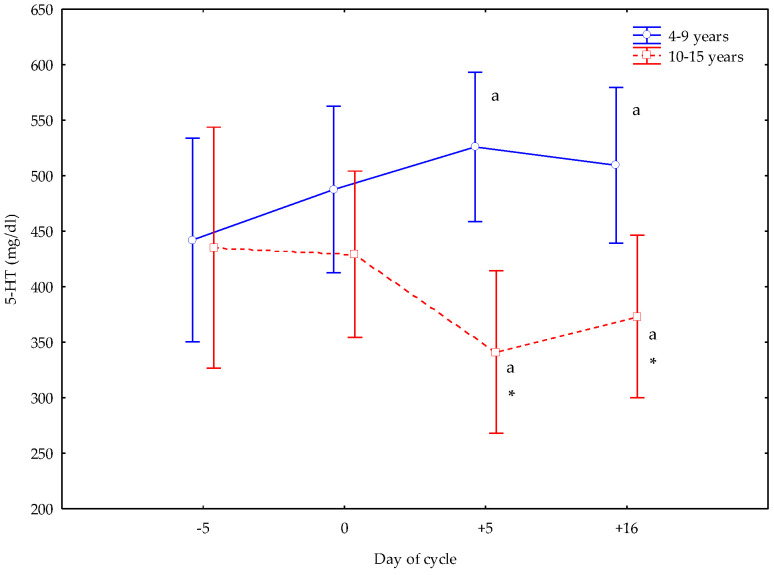
Circulating 5-hydroxytryptamine (5-HT) concentrations (Mean ± S.D.) in cyclic Spanish Purebred mares aged 4–9 years and 10–15 years. Letter indicates significant differences (*p* < 0.05) versus −5 and 0 days. Asterisk indicates significant differences (*p* < 0.05) versus mares <10 years old.

Mares from 4 to 9 years (younger) and from 10 to 15 years (older) showed an opposite 5-HT trend at +5 and +16 d of their cycle, with greater concentrations in younger mares (*p* < 0.05), and lower values in older mares (*p* < 0.05) than at −5 and 0 d. Older mares showed lower 5-HT concentrations at +5 and +16 d (*p* < 0.05) than younger mares (Figure 1).

**Figure 2:**
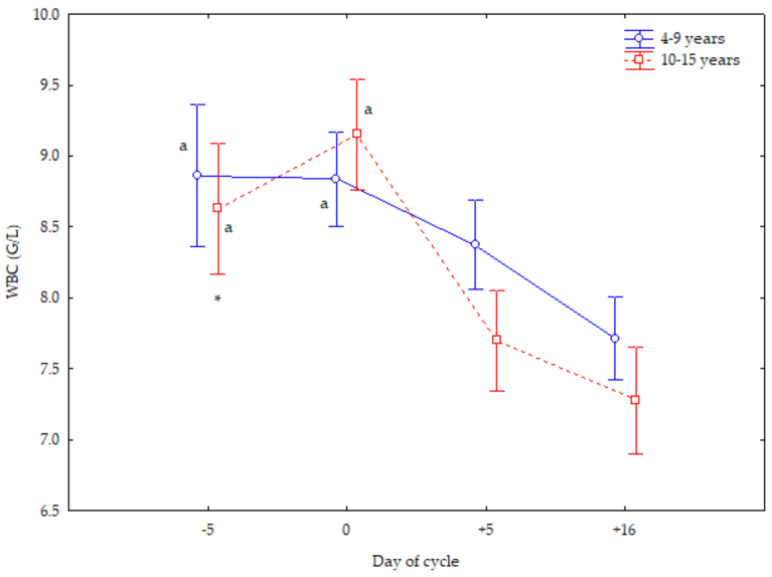
White blood cell (WBC) counts (Mean ± S.D.) in cyclic Spanish Purebred mares aged 4–9 years and 10–15 years. Letter indicates significant differences (*p* < 0.05) versus −5 and 0 days. Asterisk indicates significant differences (*p* < 0.05) versus mares <10 years old.

Mares of both ages showed a superimposed WBC trend, with the greatest number both at −5 and 0 d (*p* < 0.05). Older mares showed lower WBC numbers at −5 d (*p* < 0.05) than younger mares (Figure 2).

**Figure 3:**
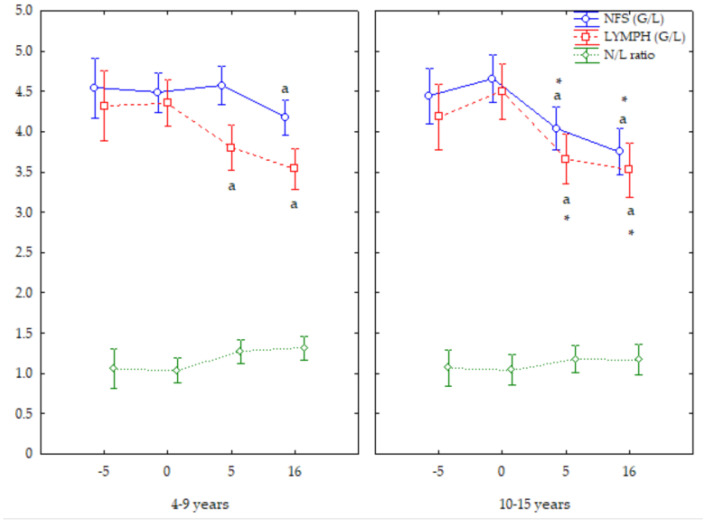
Neutrophil (NFS) and lymphocyte (LYMPH) counts and N/L ratio (Mean ± S.D.) in cyclic Spanish Purebred mares aged 4–9 years and 10–15 years. Letter indicates significant differences (*p* < 0.05) versus other days. Asterisk indicates significant differences (*p* < 0.05) versus mares <10 years old.

Older mares showed the lowest NFS and LYMPH numbers both at +5 and +16 d (*p* < 0.05); the younger mares showed the lowest NFS numbers at +16 d, and LYMPH numbers at +5 and +16 d. Older mares showed lower NFS and LYMPH numbers at both +5 and +16 d (*p* < 0.05) than younger mares. Mares of both ages showed a similar trend for the N/L ratio (Figure 3).

**Figure 4:**
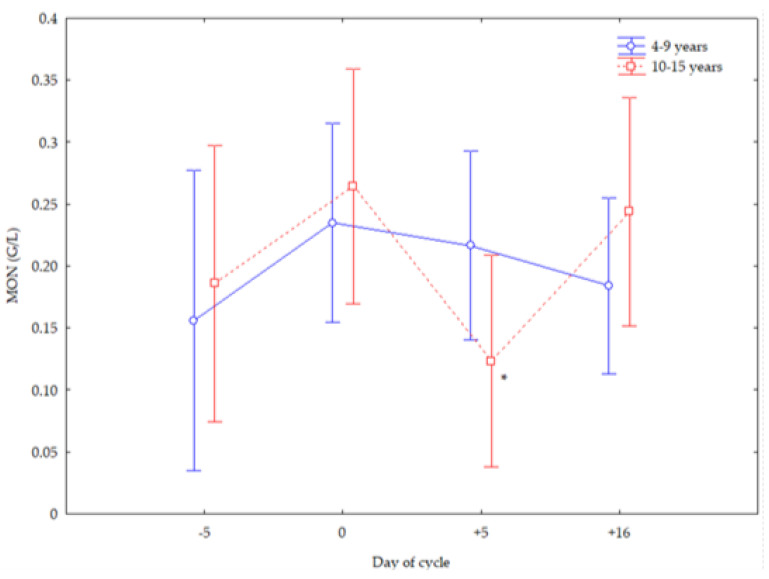
Monocyte (MON) counts (Mean ± S.D.) in cyclic Spanish Purebred mares 4–9 years and 10–15. Asterisk indicates significant differences (*p* < 0.05) versus mares <10 years old.

**Figure 5:**
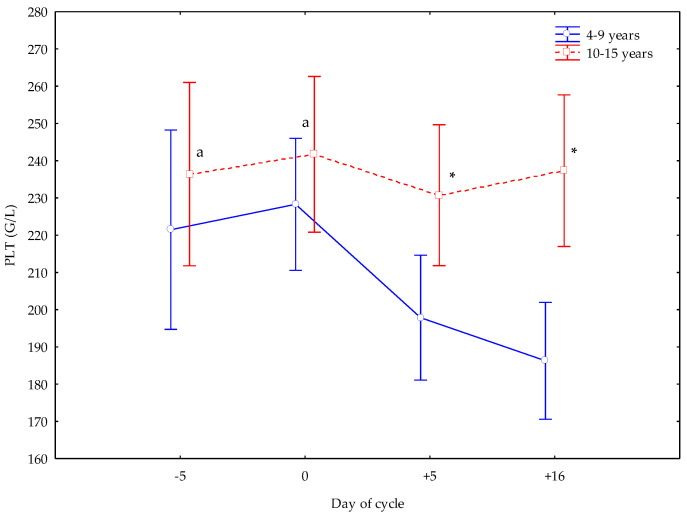
Platelet (PLT) counts (Mean ± S.D.) in cyclic Spanish Purebred mares aged 4–9 years and 10–15 years. Letter indicates significant differences (*p* < 0.05) versus +5 and +16 days. Asterisk indicates significant differences (*p* < 0.05) versus mares <10 years old.

Older mares showed lower MON numbers at +5 d (*p* < 0.05) (Figure 4), greater PLTs at +5 and +16 d (*p* < 0.05) than younger mares, and greater PLT numbers at −5 and 0 d than at +5 and +16 d (*p* < 0.05) (Figure 5).

No significant differences were observed for EOS numbers. The concentration of 5-HT was negatively correlated with LYMPHs and EOSs, and positively with WBC, NFS, MON, and PLT numbers, and with the N/L ratio (Table 1).

## 4. Discussion

Although the physiology of mares’ ovulation was extensively studied during the process of deviation, knowledge is still lacking with respect to the role of circulating 5-HT concentrations concerning pre- and post-ovulatory phases, considering its correlation with immune cell responses.

Hence, the main goal of our study was to estimate the presumed relationship between 5-HT and immune cells in healthy cyclic mares, to verify the existence of their interaction and evaluate the different trends in younger and older mares of 4–9 years and 10–15 years of age, respectively.

Circumstantial evidence suggests that, based on the biphasic and opposite 5-HT trend recorded in mares, with the greater concentrations at +5 and +16 d in younger rather than older subjects, it is possible to also consider 5-HT a regulator of cycle homeostasis in equine species, but with the lowest synthesized and secreted circulating 5-HT concentrations in old mares. What is more, the consensual lower 5-HT concentrations in the latter mares confirmed previous results described recently by Satué et al. [23] in non-pregnant mares, in which advanced age leads to reduced serotoninergic activity, presuming that synthetic molecular mechanisms are altered throughout life.

Moreover, the evolution of 5-HT concentrations observed in this study was similar to that previously shown in this same species [11,24], although the average concentrations were slightly different. In fact, foals had greater 5-HT concentrations than adult horses, indicating the highest turnover in 5-HT metabolism, probably related to nutrients and growth processes, showing that age entails a differential response in the secretion of systemic 5-HT. These conflicting data may be due to the employment of different biological samples and laboratory investigations for 5-HT assays, as they employed high-pressure liquid chromatography (HPLC) and radioimmunoassay (RIA) [25], where in our study ELISA was the technique used. What is more, other factors such as age, among others, also can influence the different ranges of this neurotransmitter [26].

Contrary to what occurs in Spanish Purebred mares, in Lusitano mares the estrous cycle did not modify the WBC count [27], as has been reported in cows [28]. However, during the estrous cycle, Kenney [29] revealed an increase in the NFS count with a tendency to migrate and accumulate in uterine venules and capillaries. This finding could suggest a privileged immune state in the uterus during the preovulatory period. Considering uterine migration, it is reasonable to think that NFSs in the blood are reduced during the follicular phase, and this could probably explain the tendency towards a higher NFS count on days +5 and +16 in the mare, as occurs in humans during the luteal phase [13,14,15,16]. This decrease in NFSs in the follicular phase could be related to the effects of estrogens since this hormone is a negative regulator of myelopoiesis in the bone marrow [30]. However, both hormones, progesterone (P4) and estrogen, increase the number of granulocytes in the bone marrow [31]. Through binding their specific receptors expressed by immune cells and/or by acting via mediators, these hormones support ovulation and the preparation of the uterus for pregnancy, although the effects of these hormones remain controversial. Some studies have shown a reduction in NFSs during the first half of diestrus, related to increased P_4_ and minimal estrogen concentrations [32]. Other studies have shown an increase in NFS chemotactic activity in response to P_4_ and a reduction in the estrogenic response [33]. However, in bitches [18], no changes in NFSs during the estrous cycle were found.

Synchronously with the decrease in 5-HT concentrations, the LYMPH and NFS counts also decreased with age in mares. It is well established that advancing age leads to a state of immunosenescence, characterized by a decrease in various types of LYMPHs [34]. There is an age-related decline in the absolute number of CD2, T, CD4+, and CD8+ LYMPHs, B LYMPHs, and NK cells, with no differences in the CD4/CD8 ratio [35]. These changes reflect the decreased production of LYMPHs by the mare’s bones and the increased exposure to antigens over time. In the same way, the proliferative response of NFS precursor cells to granulocyte-colony-stimulating factor (G-CSF) and, consequently, the NSF count in blood and bone marrow precursors decreases with age [36]. Despite this, in Andalusian broodmares of the Carthusian strain, both NFSs and the N/L ratio increased [37], while they did not change in Spanish Purebred mares with advancing age [12].

Regarding the cycle, LYMPH counts in mares were reduced in the luteal period (days +5 and +16) compared to the follicular phase. Similar results have been found in women, where the percentages of CD3+ and CD3+CD4+ T cells in peripheral blood are reduced during the mid- and late luteal phases compared to the early follicular phase [38]. This finding in women suggests a possible immunoregulatory role of P_4_ and/or estrogens on T cells, which is crucial for ovulation, embryo implantation, and corpus luteum (CL) development. Estrogen exerts immune regulation via estrogen receptors (ERs) on the LYMPHs, although receptors of P4, androgen, and glucocorticoids are found in lymphoid organs and/or LYMPHs [39].

In addition, it has been shown that P_4_ can suppress Th1 immune responses and enhance Th2 cytokine production in vitro [40]. Although LYMPH subpopulations were not determined in this study, and we can only compare the total number of peripheral LYMPHs at specific periods of the cycle, the results point to a state of immunosuppression during the luteal period, mediated by P4 and its receptors, as occurs in women. These immunosuppressive mechanisms have also been reported in women during pregnancy. In fact, P4 suppresses T cell immune functions through a non-genomic mechanism resulting from a K+ channel blockade and Ca^2+^ signaling from activated T cells, which is driven by gene expression [41]. This suppressive state is important for embryo acceptance in the event of fertilization, since the immunological environment is more tolerant to embryo development. However, other studies in humans [42] and in bitches [18] found no changes in LYMPHs during the cycle, although these results contrast with those reported in cows [43] and women [13,14,15,16], where an increase in LYMPHs has been reported along the luteal phase compared to the follicular phase, which is simultaneously accompanied by an increase in NFSs at the end of the follicular phase, before ovulation. These fluctuations in blood LYMPH and NFS counts correspond to the period of follicular atresia at the end of the luteal phase or to the development of a dominant follicle with a high concentration of P_4_ in the follicular fluid at the end of the estrous cycle [43]. The requirement for WBCs during the ovulatory process is a well-established fact; indeed, in perfused rat ovaries it has been observed that by adding peripheral blood WBCs from male rats to the perfusion system at the same concentration as in the circulation, the ovulatory response increased three-fold compared to perfusions that did not contain WBCs [44]. In cows, the LYMPH number simultaneously increases in the blood and blood vessels of the reproductive organs three or four days before ovulation [45,46].

However, during the luteal phase, changes in the populations of EOSs, NFSs, and T LYMPHs occur at critical functional stages of the CL, as shown in cows. The bovine luteolytic cascade appears to be like that of general acute inflammation in terms of time-dependent infiltration by immune cells (NFSs, MACs and T LYMPHs) [47]. In addition to their role in facilitating ovulation, immune cells may have an important role in luteal function in that the cytokines secreted by immune cells modulate both luteotropic and luteolytic processes [48].

In the mare, 5-HT concentrations were negatively correlated with LYMPHs and EOSs, and positively correlated with NFSs and the N/L ratio. Regarding LYMPHs, it has been reported that there are 5-HT receptors in LYMPHs, specifically, the receptor 5-HT_1A_ [49,50]. Although the role of 5-HT_1A_ in immune cells is still not known, it has been suggested that it is involved in cell proliferation and activation. This evidence does not appear to be present in mares, as the relationship between the 5-HT concentration and LYMPH count inversely occurs. Although future studies are needed to elucidate these mechanisms, these results seem to indicate that the circulating 5-HT concentration is not an inducer of LYMPH proliferation. Instead, a state of immunosuppression, based on a decrease in these cells, occurs when 5-HT increases, particularly in the luteal phase of the cycle.

Regarding NFSs, it has been shown that 5-HT enhances their recruitment to sites of inflammation, and there was less NFS recruitment in the absence of 5-HT [51,52], which could affect the inflammatory response present during a normal estrous cycle, although these mechanisms remain unknown in the mare.

The MON count decreased with advancing age in the mares. It is known that antigen-specific immune responses or adaptive immunity can be altered. Indeed, advanced age leads to a state of chronic low-grade inflammation that contributes to the pathogenesis of many related diseases in elderly individuals. This inflammatory state is associated with chronic activation of the innate immune system, characterized by elevated concentrations of circulating proinflammatory cytokines, such as TNF, IL-6, and IL-8 [53]. Compared to older mares, younger ones showed greater MON numbers at +5 d; this luteal increase in MONs confirms the increase in the percentage of peripheral MONs producing TNF-α and IL-1β in vitro in women, after stimulation with endotoxin, compared to the follicular phase of the ovarian cycle [9,54]. These cellular changes have been related to the increase in P4 and estradiol 17-β (E2) during the luteal phase. However, no changes in MONs were reported in bitches during their cycle [18]. The positive correlation between 5-HT and MONs seems expected since some human studies have reported that 5-HT activates MONs, preventing their apoptosis. In fact, normal MONs treated with 5-HT showed increased expression of costimulatory molecules and a greater capacity to produce cytokines after treatment with lipopolysaccharides [55].

Although age did not modify the count in this study, a previous study in mares revealed increased EOS counts along the lifespan [37]. This evidence was confirmed by Willson et al. [56], who reported 45.8% more circulating EOSs in the diestrus bitch.

PLTs are an integral carrier of 5-HT in the periphery, as they transport 5-HT released from enterochromaffin cells lining the gut into the bloodstream. The relationship between PLTs and 5-HT is bidirectional: on one side, 5-HT plays a role in the activation and aggregation of PLTs; on the other side, PLTs include a great amount of 5-HT in their dense granules and express 5-HT receptors (5-HT2A) and transporters on the cell surface [8,57]. Indeed, PLTs store 5-HT at very high concentrations and secrete it upon activation [8], which explains the close relationship between both parameters, as has been documented in horses [26,58]. Although in this study the PLT counts did not change, previous studies in this same species showed a decrease in PLTs with advanced age [58]. Diverse studies have reported a lower PLT count in older individuals. Although the mechanisms underlying their count decline during aging are unknown, it has been suggested that a reduction in the megakaryocytic stem cell pool could be responsible for the decrease in PLT production [59,60]. On the contrary, other studies found no significant changes in the leukogram in bitches during the estrous cycle [17,61].

## 5. Conclusions

Advancing age leads to a minor plasma 5-HT concentration in the cyclic mare. This 5-HT pattern could suggest a reduced serotoninergic activity in the oldest mares, as moreover confirmed by the existence of a negative and significant correlation between 5-HT and aging. The results obtained in the present study indicate that the activity and the effect of 5-HT display a specific temporal pattern according to different ages. This finding suggests that the reductions in 5-HT synthesis and release and the negative correlations between it and age could be due to a different molecular mechanism of this neurotransmitter with advancing age.

Moreover, the significant 5-HT decrease during the post-ovulation days, described only in older animals, could be an expression of a defensive strategy in the mares, with implementation of negative feedback on 5-HT, as described in women [62]. However, although in women 5-HT receptors are broadly expressed in reproductive tissues [62], detailed data and studies do not exist in the equine species; hence, in light of the similarities in their reproductive physiology [63,64], some extrapolation could also be made for the involvement of 5-HT during pre- and post-ovulatory timeframes on immune responses and inflammation.

## Figures and Tables

**Table 1:** Correlations among 5-hydroxytryptamine (5-HT) concentrations and the number of white blood cells (WBCs), neutrophils (NFSs), lymphocytes (LYMPHs), eosinophils (EOSs), monocytes (MONs), and platelets (PLTs).

	WBCs (G/L)	LYMPHs (G/L)	NFSs (G/L)	N/L Ratio	EOSs (G/L)	MONs (G/L)	PLTs (G/L)
5-HT (ng/mL)	0.20	−0.13	0.47	0.34	−0.06	0.15	0.15
WBCs (G/L)		0.75	0.61	−0.24	0.33	0.30	0.26
LYMPHs (G/L)			−0.05	−0.77	0.41	0.24	0.28
NFSs (G/L)				0.56	0.01	0.16	0.07
N/L ratio					−0.17	−0.10	−0.23
EOSs (G/L)						−0.01	−0.12
MONs (G/L)							−0.07

## Data Availability

The data that support this study will be shared upon reasonable request to be corresponding author.
